# Supplementary material for: Cholesterol restriction primes antiviral innate immunity via SREBP1-driven noncanonical type I IFNs
Source: EMBO Rep. 2024 Dec 12;26(2):560–92. doi: 10.1038/s44319-024-00346-9 (PMC11772592; doi:10.1038/s44319-024-00346-9)
Supplement: Supplementary file 1 — Appendix [file 44319_2024_346_MOESM1_ESM.pdf]

Appendix for:

Cholesterol Restriction Primes Antiviral Innate Immunity via SREBP1-Driven Noncanonical Type I IFNs

<u>Table of Content</u>

Face Page and Table of Contents	Page 1
Appendix Figure S1	Page 2
Appendix Figure S2	Page 3
Appendix Figure S3	Page 4
Appendix Figure S4	Page 5
Appendix Figure S5	Page 6
Appendix Figure S6	Page 7
Appendix Figure S7	Page 8
Appendix Figure S8	Page 9
Appendix Tables S1	Page 10

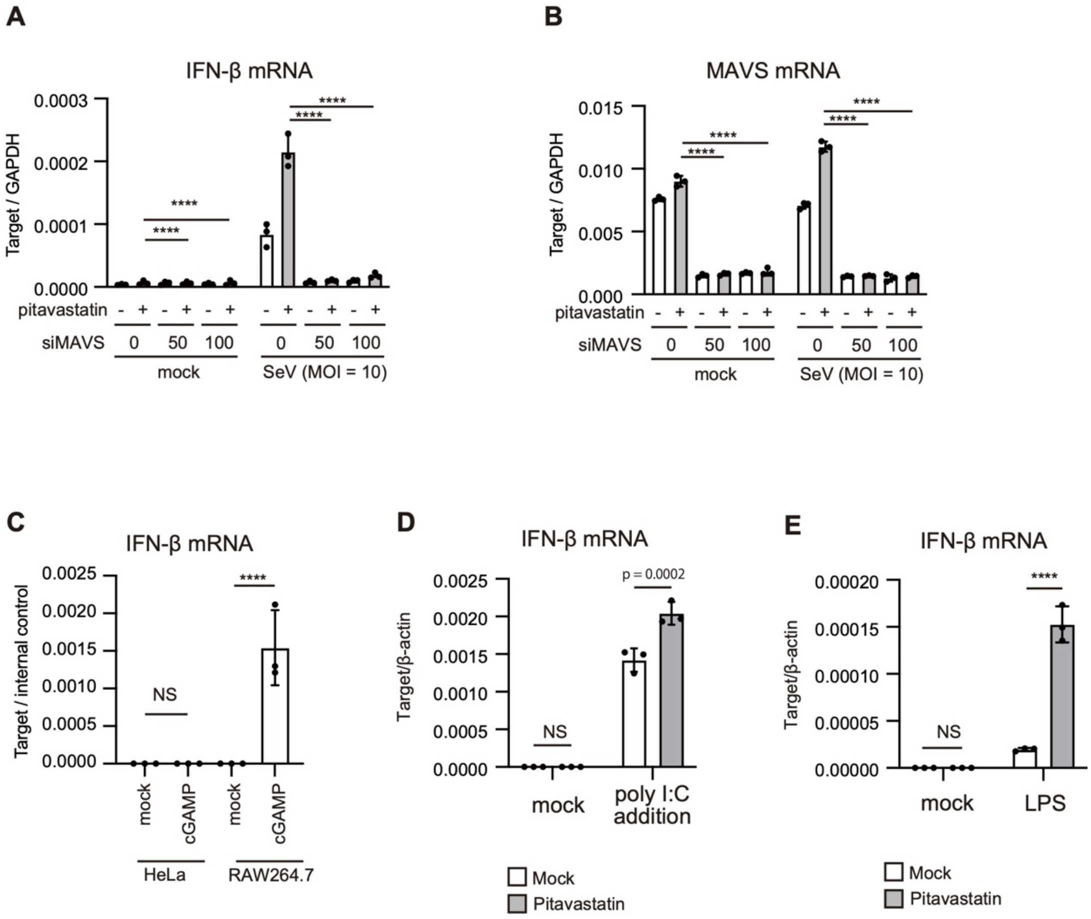

Appendix Figure S1. Effects of PAMPs

A, B) HeLa cells were transfected with siRNA for MAVS at indicated concentrations for 2 days. Cells were treated with 2 µM of pitavastatin for 24 h and then infected with MOI = 10 for 24 h. The expression of IFN-β (A) and MAVS (B) were determined by RT-qPCR and normalized to GAPDH.

C) HeLa and RAW264.7 cells were stimulated by transfecting cGAMP (8 µg/mL). The expression of each gene was determined by RT-qPCR and normalized to that of GAPDH.

D) RAW264.7 cells were treated with 2 µM of pitavastatin for 24 h, and then stimulated by adding poly I:C (50 µg/ml) to the cell culture medium. 2 h after stimulation, the expression of IFN-β mRNA was measured by RT-qPCR and normalized to β-actin.

E) RAW264.7 cells were treated with 20 µM of pitavastatin for 24 h and then stimulated with LPS for 2 h. The expression of IFN-β mRNA was measured by RT-qPCR and normalized to β-actin.

Data Information: In (A–E), data are represented as the mean ± SD. Statistical significance was determined using two-way ANOVA. ****$p < 0.0001$. NS: not significant.

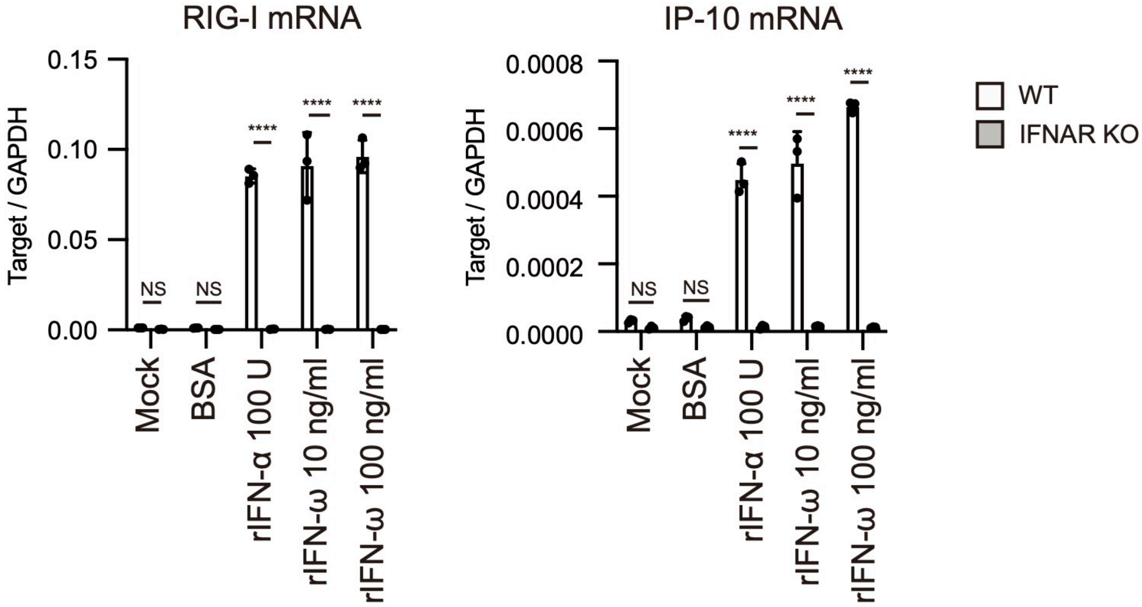

Appendix Figure S2. Confirmation of IFNAR2 KO cells

WT and IFNAR2 KO cells were stimulated with recombinant IFN-α and IFN-ω, and then the expression of RIG-I and IP10 mRNA levels were determined by RT-qPCR and normalized to GAPDH. IFNAR2 KO cells did not express RIG-I and IP-10 mRNA even after type I IFN stimulation. These data confirmed that IFNAR2 KO cells do not respond to type I IFNs.

Data Information: data are represented as mean ± SD. Statistical significance was determined using two-way ANOVA. ****$p < 0.0001$. NS: not significant.

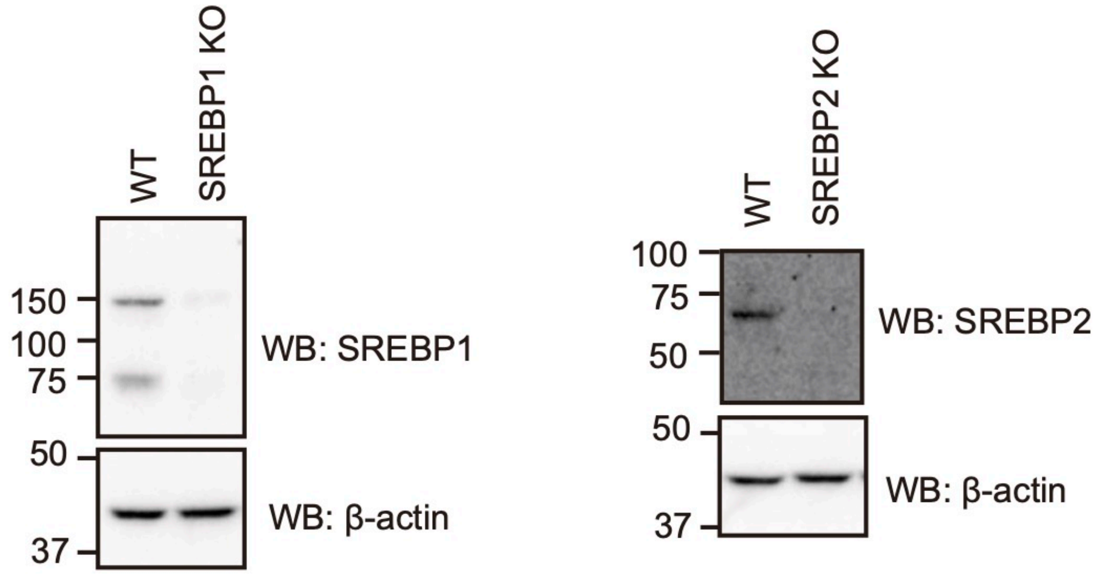

Appendix Figure S3. Knockout of SREBP1 and 2

Whole cell lysates from WT and SREBP1 and 2 KO HeLa cells were prepared and subjected to SDS-PAGE. The proteins were detected by Western blotting using the indicated antibody. The data indicate that each KO cell line lacks the expression of the SREBP1 or 2 proteins.

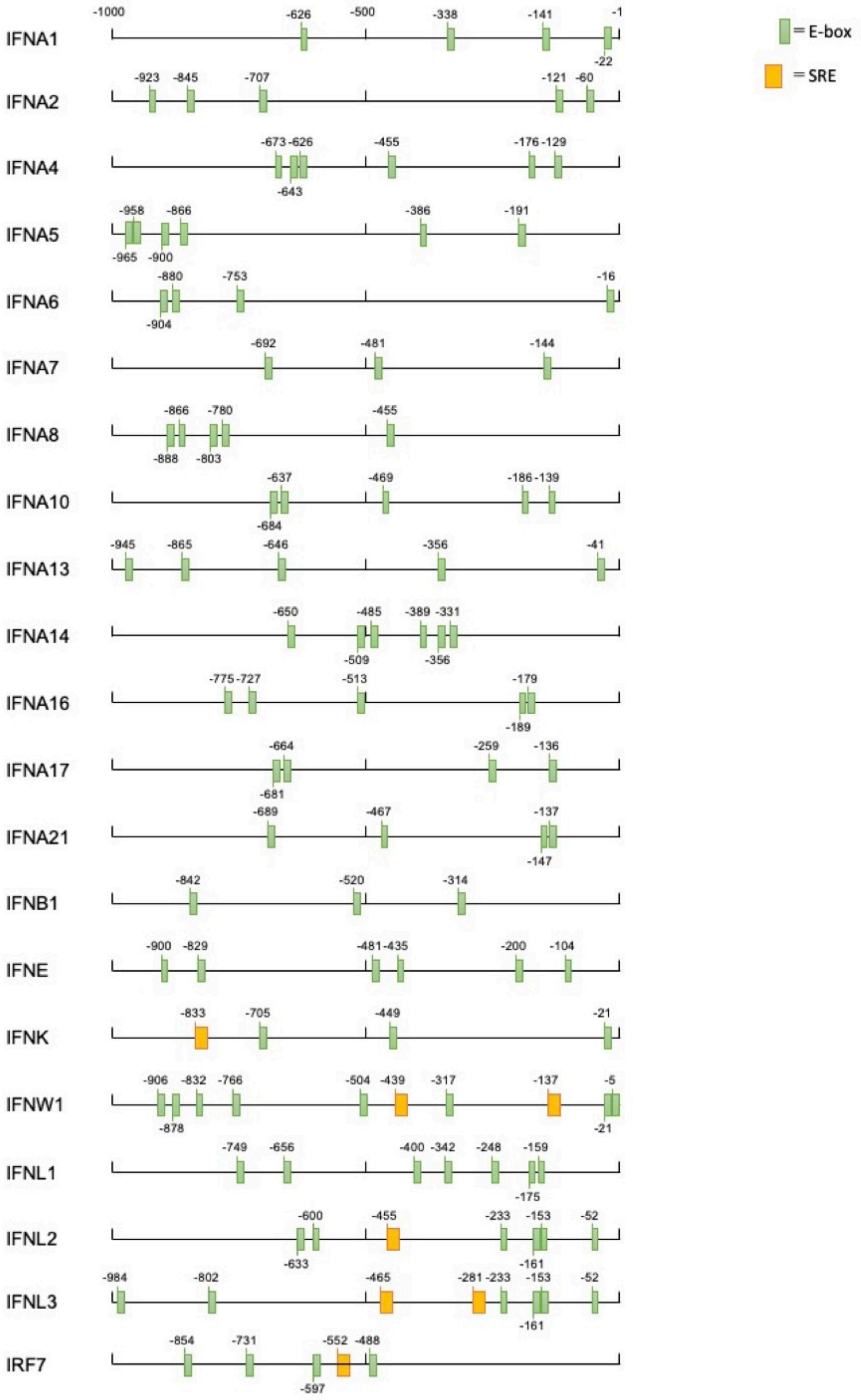

Appendix Figure S4. SRE elements and E-boxes in the promoters

The E-boxes and SRE elements on the promoter regions of human type I IFNs, type III IFNs, and IRF7 genes are depicted.

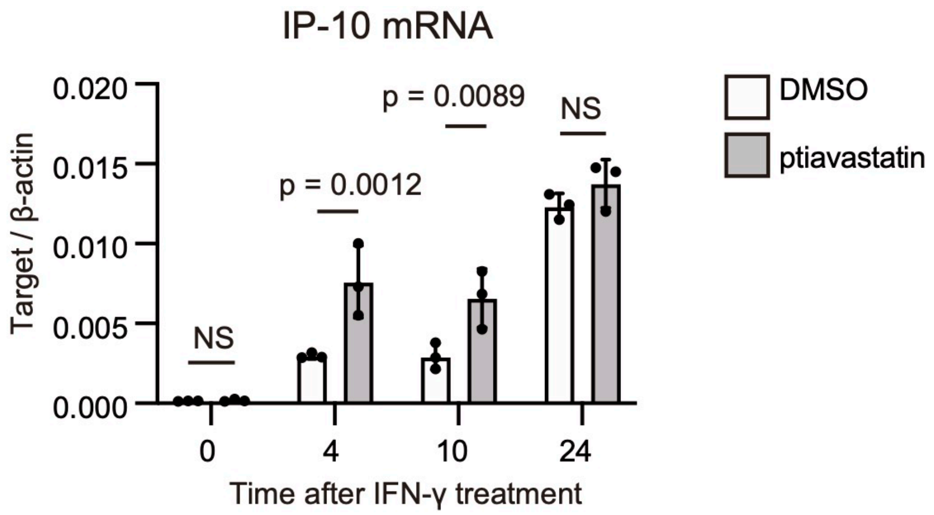

Appendix Figure S5. Effects of pitavastatin on IFN-γ-mediated chemokine expression

RAW264.7 cells were treated with 10 μM of pitavastatin for 24 h. Cells were then stimulated with 40 ng/ml of IFN-γ at indicated hours. The expression of IP10 was measured by RT-qPCR and normalized to β-actin.

Data Information: data are presented as mean ± SD. Statistical significance was determined by two-way ANOVA. NS: not significant.

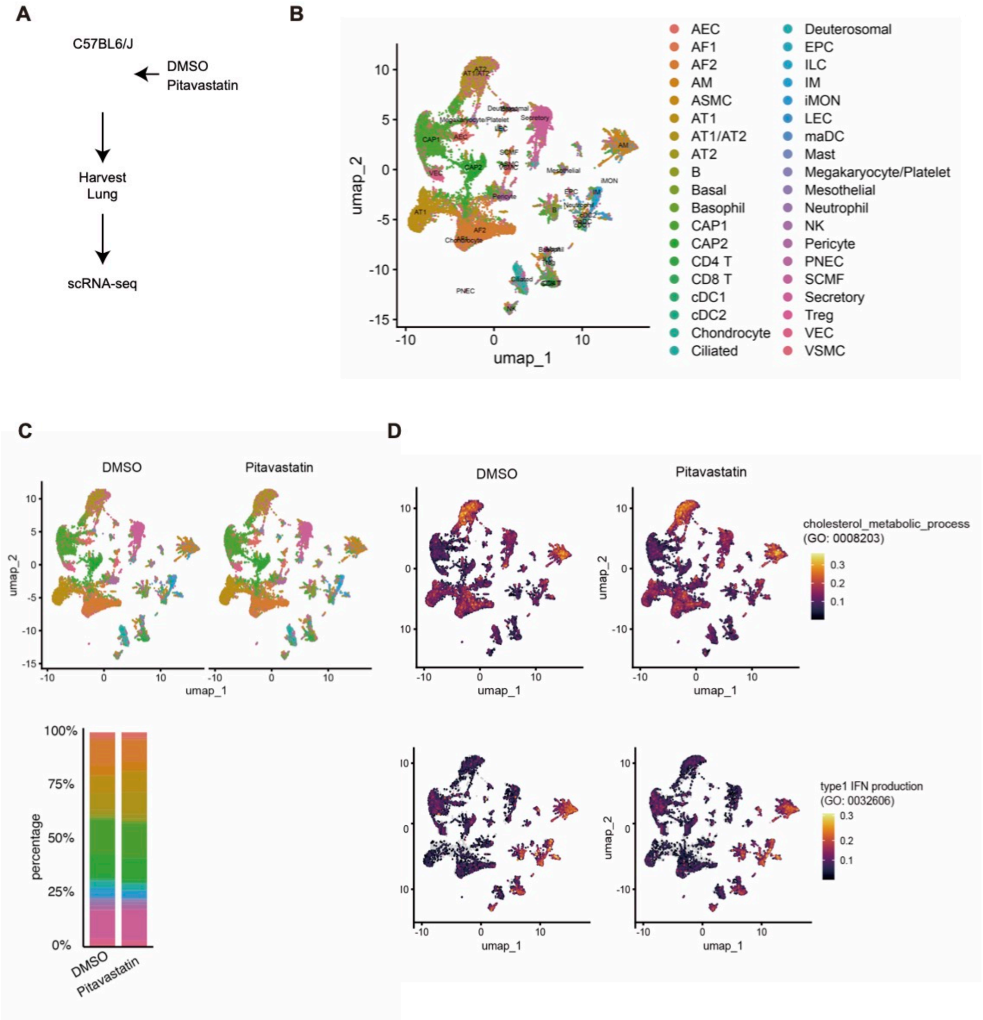

Appendix Figure S6. Single-cell RNA-seq analysis of mouse lungs following pitavastatin administration

A) Schematic representation of the single-cell analysis. Mice were injected with pitavastatin or solvent (DMSO) every three days. The lung tissues were harvested and subjected to single-cell RNA-Seq analysis.

B) UMAP visualization of total lung cell populations identified in the scRNA-seq data. Each dot represents a single cell, with colors indicating different cell types described in the legend to the right of the figure.

C) UMAP plots comparing the distribution of lung cell populations between pitavastatin and DMSO-injected mice. The bottom bar graph shows the percentage of each cell type in the respective groups.

D) Functional annotation of gene expression profiles in lung cells from DMSO and pitavastatin-injected mice. The top UMAP plots highlight cells with expressing genes related to the cholesterol metabolic process (GO: 0008203), while the bottom plots show cells with expressing genes related to type I IFN production (GO: 0032606). The intensity of color intensity corresponds to the expression level of the gene associated with the respective biological process.

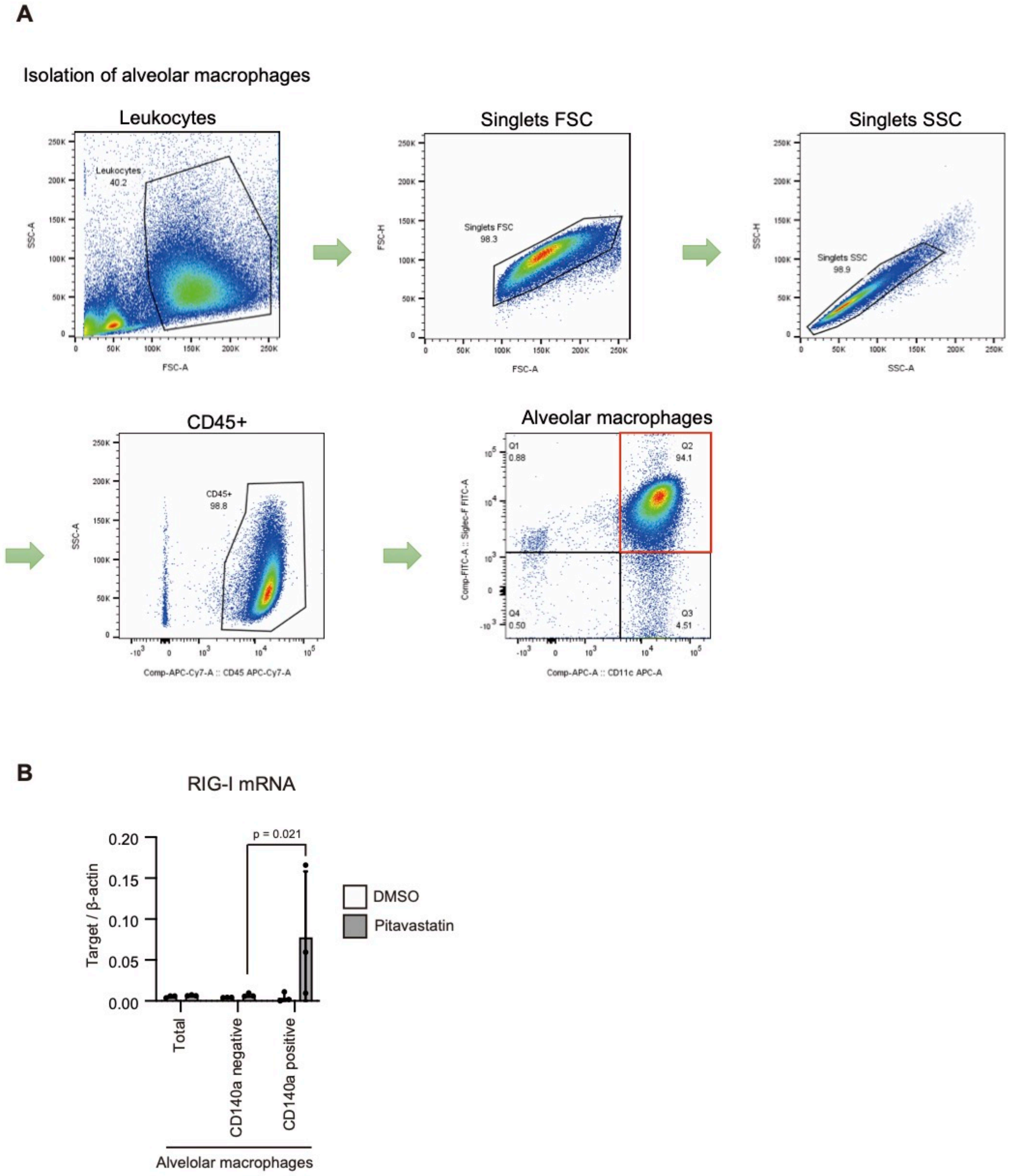

Appendix Figure S7. Gating strategy for the isolation of alveolar macrophages

A) Cells were isolated from wild-type mice lungs. The gating strategies for the identification of alveolar macrophages were shown.

B) CD140a-positive alveolar macrophages were isolated from the lung using a MACS column, and cells were treated with DMSO or pitavastatin (2 µM) for 24 h. The expression of RIG-I mRNA was compared among CD140a positive, CD140a negative (flow-through), and total (CD140a positive + CD140a negative) alveolar macrophage fractions.

In each graph, the data represents the mean ± SD. Statistical significance was determined using two-way ANOVA.

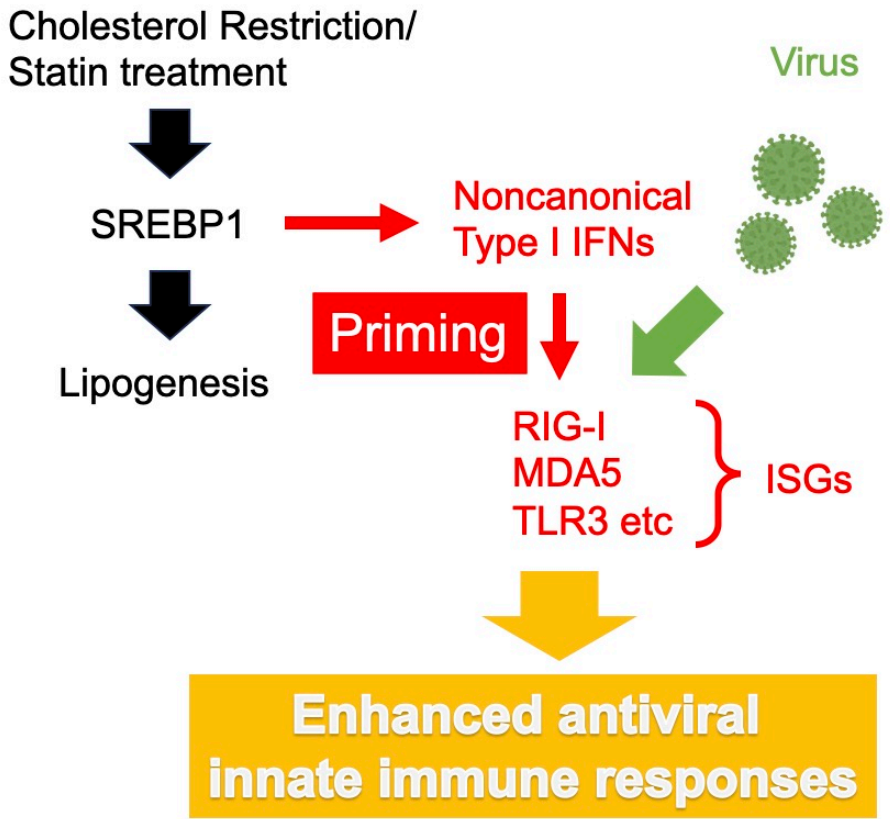

Appendix Figure S8. Mechanism of Cholesterol restriction-mediated enhancement of antiviral innate immune responses

Cholesterol synthesis restriction, induced by statin treatment, activates SREBP1, which upregulates genes not only for lipid synthesis but also for noncanonical type I IFNs. These IFNs promote the expression of RIG-I, MDA5, and TLR3, enhancing antiviral innate immune responses.

Cluster	PR3644_01	PR3644_02	Total
0	88	386	474
1	257	174	431
2	269	75	344
3	51	98	149
4	58	82	140
5	42	57	99
6	27	42	69
7	38	15	53
8	13	26	39
9	12	27	39
10	10	17	27

Appendix Table S1. The number of cells for violin plots in Figure 6
