# Supplementary figures and images for: Cholesterol restriction primes antiviral innate immunity via SREBP1-driven noncanonical type I IFNs
Source: EMBO Rep. 2024 Dec 12;26(2):560–92. doi: 10.1038/s44319-024-00346-9 (PMC11772592; doi:10.1038/s44319-024-00346-9)

## Slide 1
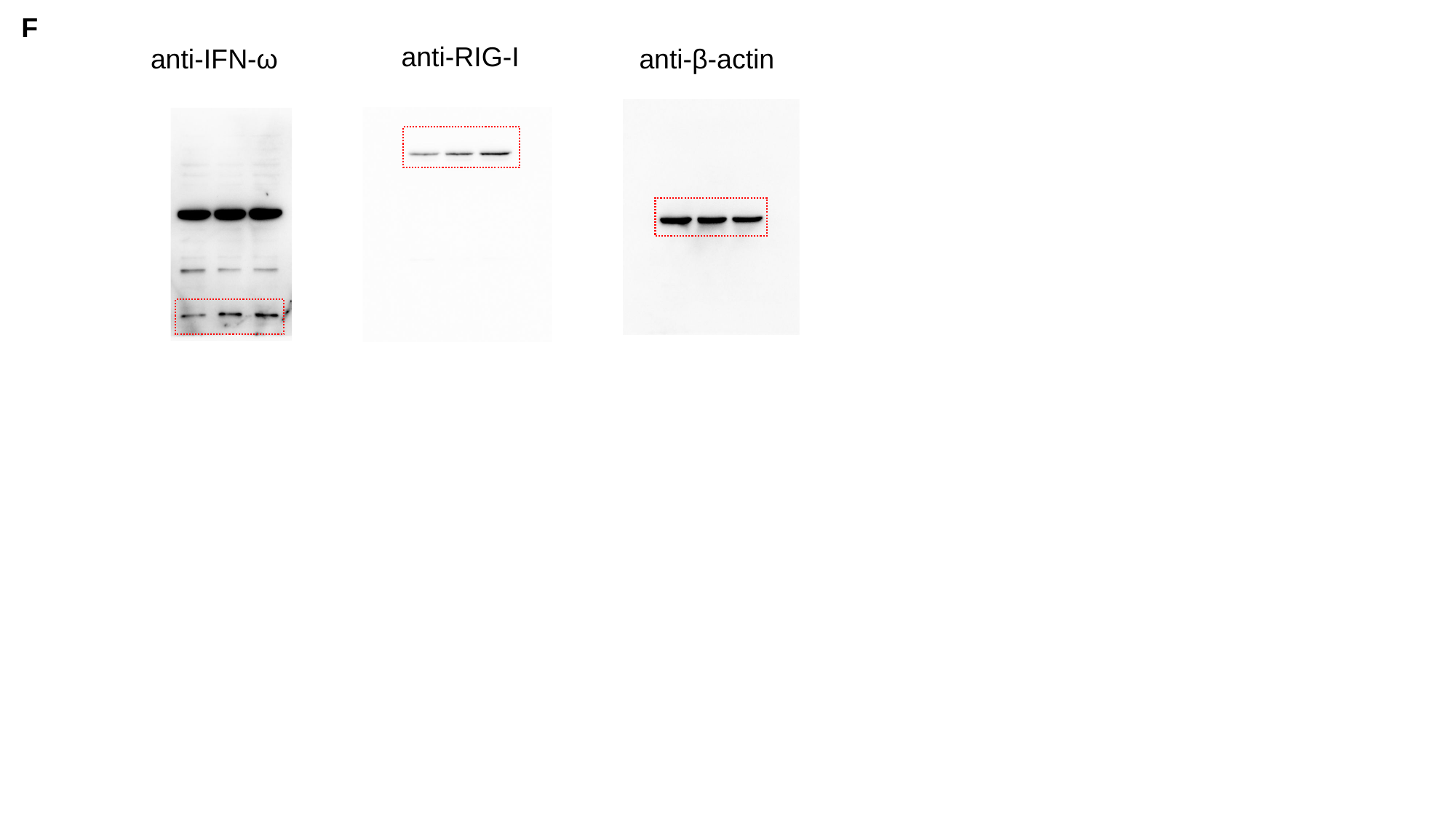

F
anti-RIG-I
anti-IFN-ω
anti-β-actin

Supplement: Supplementary file 4 — Source data Fig. 2 [file 44319_2024_346_MOESM4_ESM.zip › Figure 2/Fig 2F.pptx]

## Slide 1
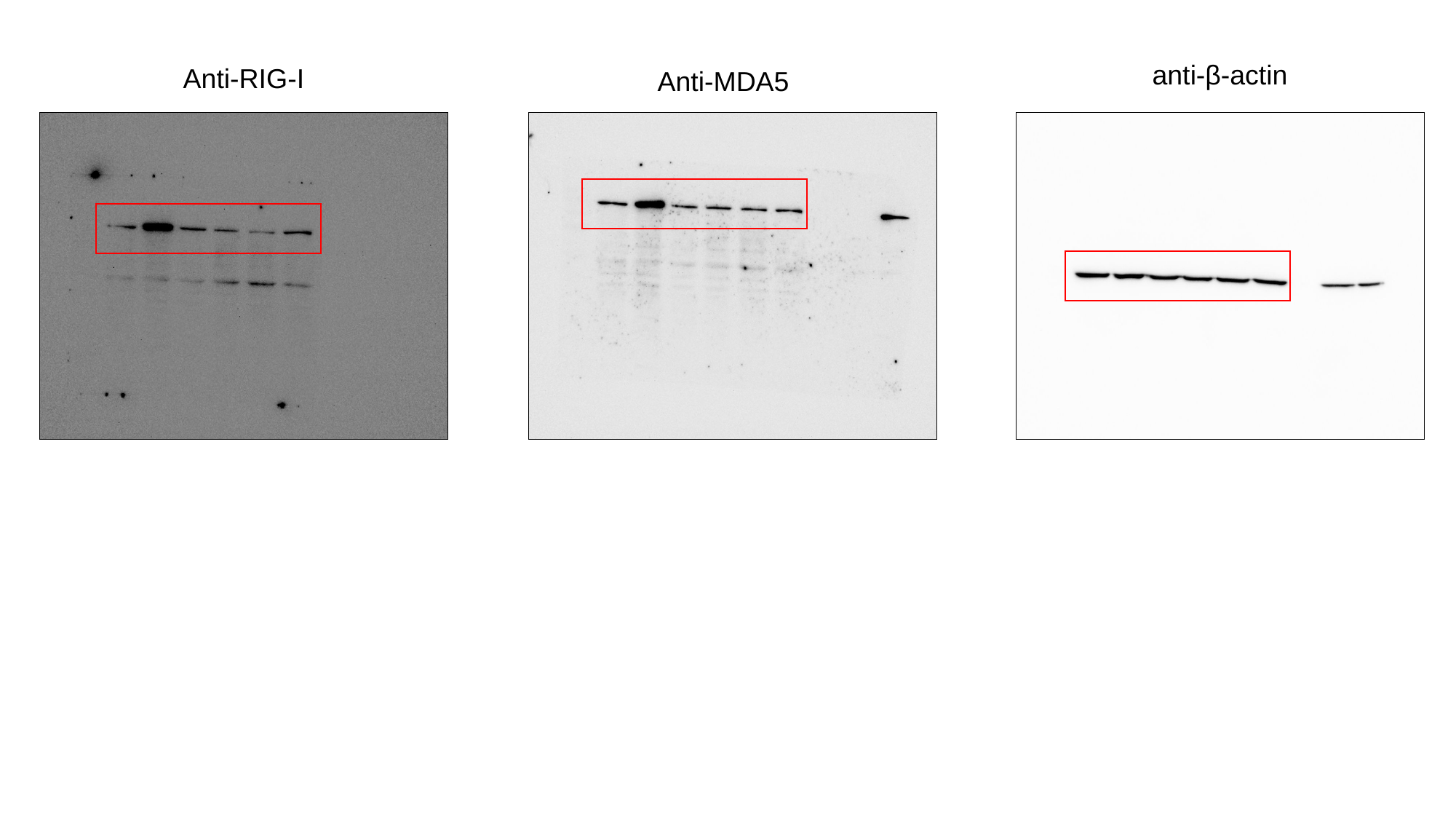

anti-β-actin
Anti-RIG-I
Anti-MDA5

Supplement: Supplementary file 5 — Source data Fig. 3 [file 44319_2024_346_MOESM5_ESM.zip › Figure 3/Fig 3L.pptx]
