# Supplementary material for: Cholesterol restriction primes antiviral innate immunity via SREBP1-driven noncanonical type I IFNs
Source: EMBO Rep. 2024 Dec 12;26(2):560–92. doi: 10.1038/s44319-024-00346-9 (PMC11772592; doi:10.1038/s44319-024-00346-9)
Supplement: Supplementary file 9 — Expanded View Figures [file 44319_2024_346_MOESM9_ESM.pdf]

Expanded View Figures

Figure EV1. Effects of statins on HeLa cells.

(**A–H**) HeLa cells were treated with pitavastatin or DMSO (mock) for 24 h, Subsequently, they were stimulated by either transfecting 200 ng/mL of short poly I:C (**A**, **B**), infection with SeV (**C**) at MOI = 10 or Flu at MOI = 10 (**D**), adding poly I:C (50 μg/mL) to cell culture medium (**E–G**), or transfecting cGAMP (8 μg/mL) (**H**). siRNAs for negative control (si Con) and TLR3 (si TLR3) were transfected into HeLa cells for 2 days and then stimulated by adding poly I:C (50 μg/mL) to the cell culture medium (**G**). The expression of each gene was determined by RT-qPCR and normalized to that of GAPDH ($n = 3$, (**A–C**); technical replicates, (**D–H**); biological replicates). (**I–K**) HepG2 (**I**), A549 (**J**), and HeLa (**K**) cells were treated with pitavastatin (**I**, **J**), cerivastatin (**K**), or solvent (DMSO) for 24 h, and the cellular cholesterol levels were measured. Statin-pretreated cells were infected with SeV for 24 h, and then the expression of IFN-β mRNA was determined by RT-qPCR and normalized to that of GAPDH ($n = 3$ biological replicates). (**L**, **M**) HeLa (**L**) and HepG2 (**M**) cells were transfected with HMGCR or control siRNA. Twenty-four hours after siRNA transfection, the cells were stimulated by short poly I:C transfection (200 ng/mL) for the indicated time period. The mRNA expression of each gene was determined using RT-qPCR and normalized to that of GAPDH ($n = 3$ biological replicates). Data Information: in (**A–M**), Data are represented as mean ± SD ($n = 3$, ****$p < 0.0001$, one-way ANOVA (**I–K**), two-way ANOVA (**A–H**), NS: not significant).

 © The Author(s)

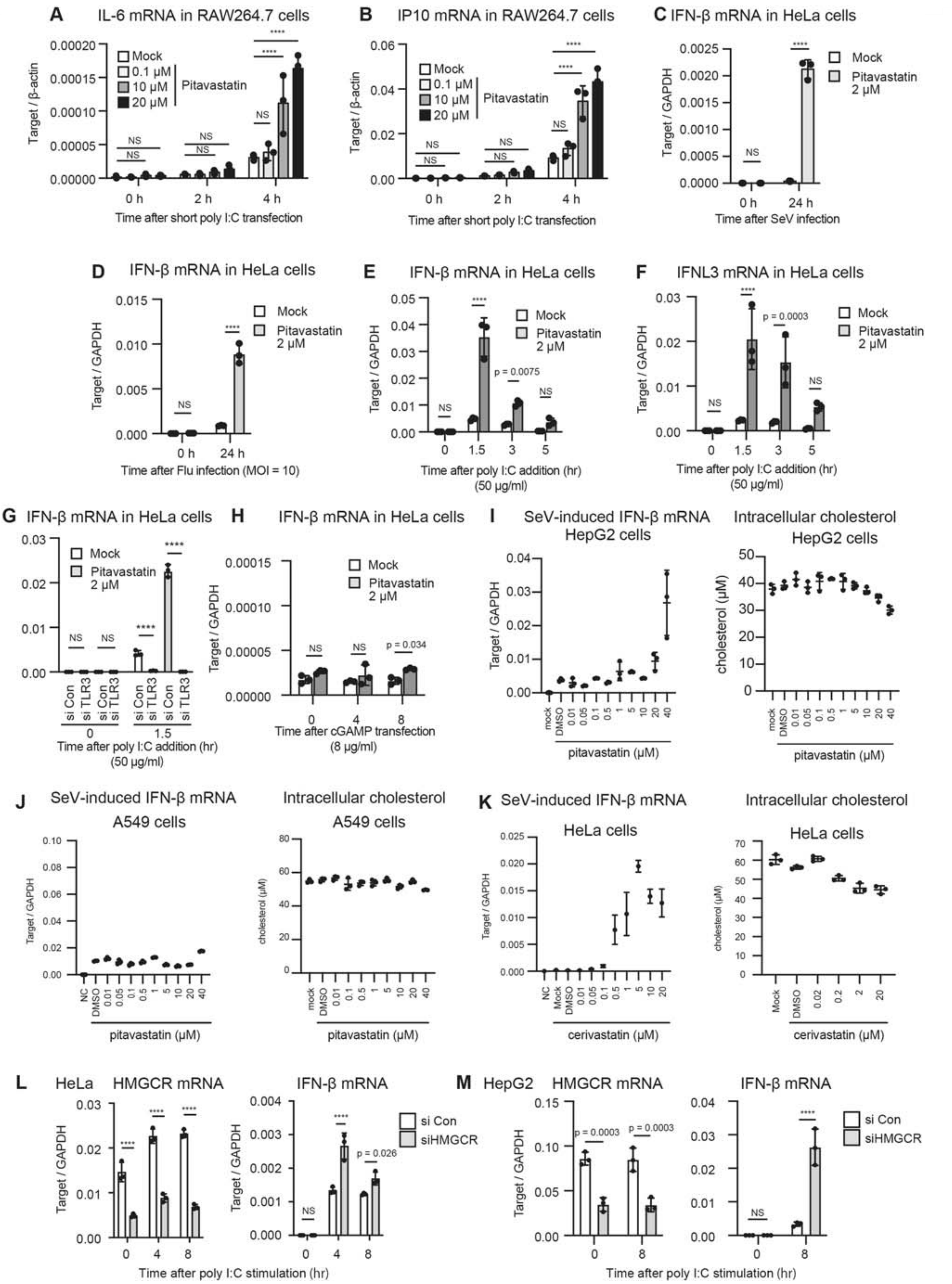
A IL-6 mRNA in RAW264.7 cells
Target / β-actin
Mock
0.1 μM
10 μM
20 μM
Pitavastatin
NS
0 h
2 h
4 h
Time after short poly I:C transfection
B IP10 mRNA in RAW264.7 cells
Target / β-actin
Time after short poly I:C transfection
C IFN-β mRNA in HeLa cells
Target / GAPDH
Mock
Pitavastatin 2 μM
0 h
24 h
Time after SeV infection
D IFN-β mRNA in HeLa cells
Time after Flu infection (MOI = 10)
E IFN-β mRNA in HeLa cells
p = 0.0075
Time after poly I:C addition (hr) (50 μg/ml)
F IFNL3 mRNA in HeLa cells
p = 0.0003
Time after poly I:C addition (hr) (50 μg/ml)
G IFN-β mRNA in HeLa cells
si Con
si TLR3
Time after poly I:C addition (hr) (50 μg/ml)
H IFN-β mRNA in HeLa cells
p = 0.034
Time after cGAMP transfection (8 μg/ml)
I SeV-induced IFN-β mRNA HepG2 cells
Intracellular cholesterol HepG2 cells
cholesterol (μM)
mock
DMSO
pitavastatin (μM)
J SeV-induced IFN-β mRNA A549 cells
Intracellular cholesterol A549 cells
NC
pitavastatin (μM)
K SeV-induced IFN-β mRNA HeLa cells
Intracellular cholesterol HeLa cells
Mock
cerivastatin (μM)
L HeLa HMGCR mRNA
IFN-β mRNA
si Con
siHMGCR
p = 0.026
Time after poly I:C stimulation (hr)
M HepG2 HMGCR mRNA
IFN-β mRNA
p = 0.0003
Time after poly I:C stimulation (hr)

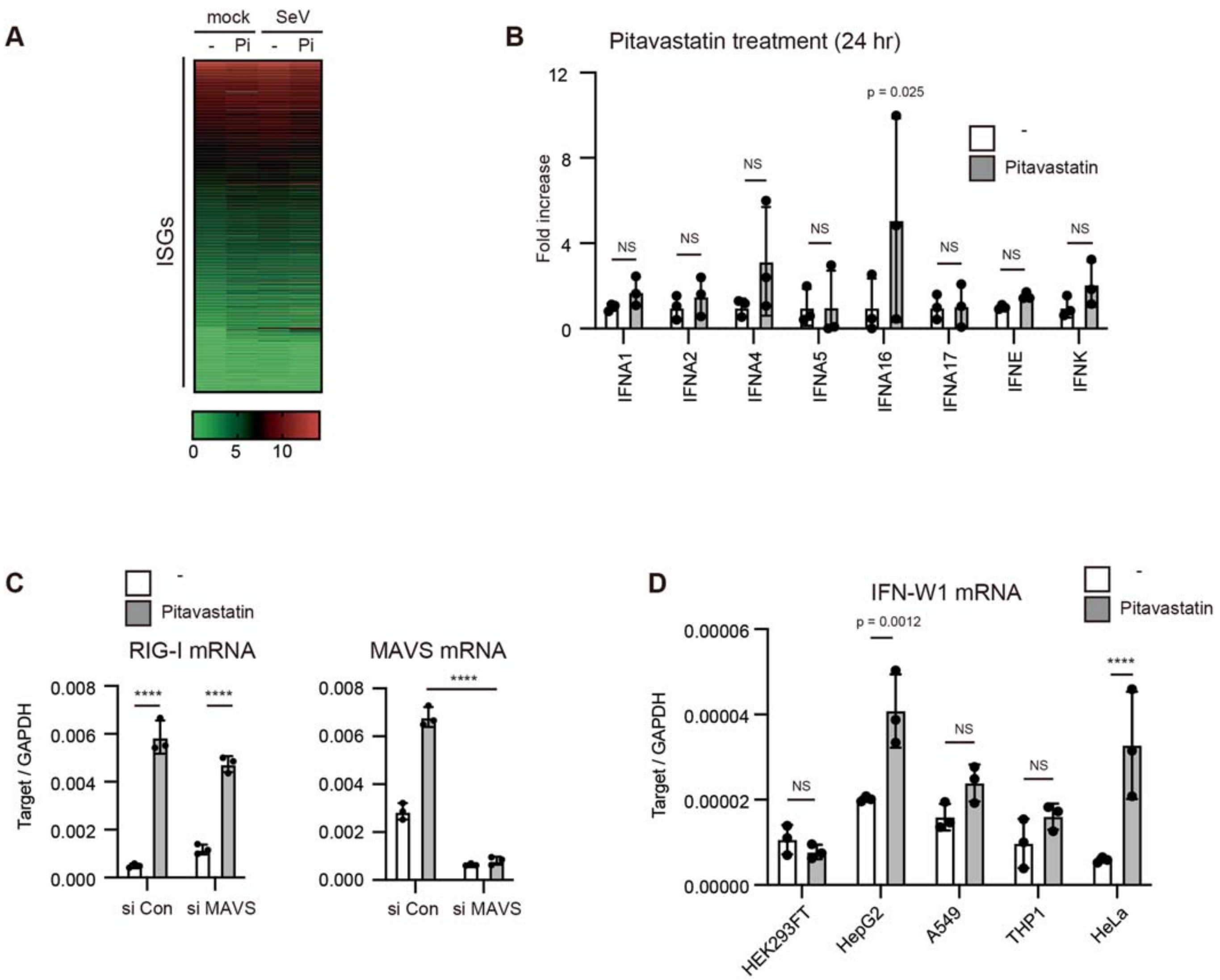

Figure EV2. Expression of non-canonical type I IFNs after pitavastatin treatment.

(**A**) Heatmap of interferon-stimulated genes (ISGs) from the microarray analysis presented in Fig. 2. ISGs were selected by searching for interferon-stimulated genes encoding proteins in the GeneCARDs database (https://www.genecards.org). (**B**) HeLa cells were treated with pitavastatin for 24 h. The expression of each gene was determined by RT-qPCR and normalized to that of β-actin. The fold increase was calculated by dividing the normalized expression level of a pitavastatin-treated sample by a pitavastatin-untreated sample value ($n = 3$, biological replicates). (**C**) HeLa cells were transfected with siRNA for MAVS (si MAVS) and negative control (si Con) for 72 h. Cells were then treated with pitavastatin for 48 h. The expression of RIG-I and MAVS were determined by RT-qPCR and normalized to GAPDH ($n = 3$, biological replicates). (**D**) HEK293, HepG2, A549, THP-1, and HeLa cells were treated with pitavastatin for 24 h, and IFN-ω expression was determined by RT-qPCR and normalized to that of GAPDH ($n = 3$, technical replicates). Data Information: In (**B–D**), data are represented as mean ± SD (****$p < 0.0001$, two-way ANOVA, NS: not significant).

 © The Author(s)

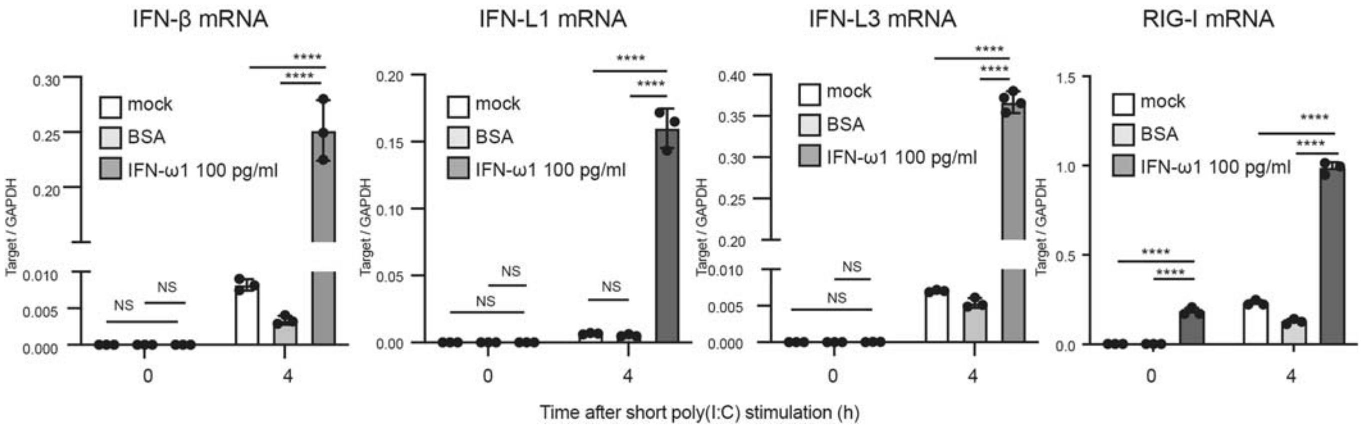

Figure EV3. Effect of IFN-ω on the RIG-I-dependent innate immune response.

HeLa cells were treated with 100 pg/mL of recombinant IFN-ω protein for 24 h, and stimulated with short poly I:C (200 ng/mL) transfection. The expression of each gene was determined by RT-qPCR and normalized to that of GAPDH. Data Information: Data are represented as mean ± SD ($n = 3$, biological replicates, ****$p < 0.0001$, two-way ANOVA, NS: not significant).

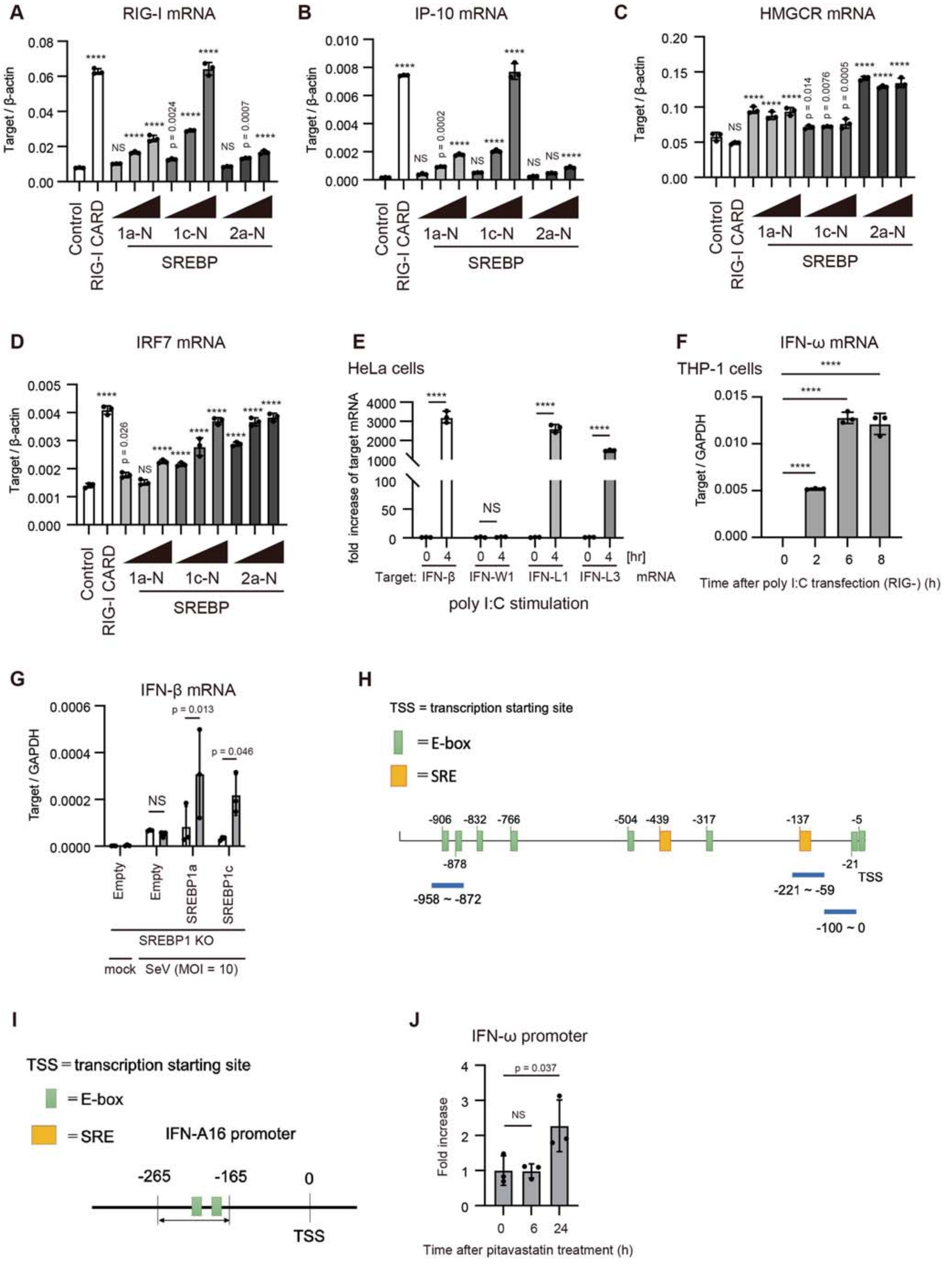
A
RIG-I mRNA
Target / β-actin
Control
RIG-I CARD
1a-N
1c-N
2a-N
SREBP
B
IP-10 mRNA
C
HMGCR mRNA
D
IRF7 mRNA
E
HeLa cells
fold increase of target mRNA
Target: IFN-β
IFN-W1
IFN-L1
IFN-L3
mRNA
poly I:C stimulation
F
IFN-ω mRNA
THP-1 cells
Target / GAPDH
Time after poly I:C transfection (RIG-) (h)
G
IFN-β mRNA
Empty
SREBP1a
SREBP1c
SREBP1 KO
mock
SeV (MOI = 10)
H
TSS = transcription starting site
=E-box
=SRE
TSS
I
IFN-A16 promoter
J
IFN-ω promoter
Fold increase
Time after pitavastatin treatment (h)

© The Author(s)

Figure EV4. The role of SREBPs in type I IFN responses.

(**A–D**) HEK293 cells were transfected with 200 ng/mL of RIG-I CARD expressing vectors or 200, 1000, or 2000 ng/mL of SREBP1a-N, 1c-N, or 2a-N expressing vectors in a 24-well plate. The total amount of DNA was adjusted to the same amount by adding an empty vector. Twenty-four hours after transfection, the expression of each gene was determined by RT-qPCR and normalized to that of β-actin. Each *p*-value represents the comparison with the control ($n = 3$, technical replicates). (**E**) HeLa cells were stimulated via transfection with 200 ng/mL of short poly I:C, and the mRNA expression of each gene was determined by RT-qPCR. The fold increase was calculated by dividing mRNA expression at each time point by that at 0 h ($n = 3$, biological replicates). (**F**) THP1 cells were stimulated via transfection with short poly I:C, and the expression of IFN-ω mRNA was determined by RT-qPCR and normalized to that of GAPDH ($n = 3$, technical replicates). (**G**) SREBP1 KO HeLa cells were transfected with empty vector (Empty) or full-length SREBP1a or SREBP1c. Twenty-four hours after transfection, cells were treated with pitavastatin for 24 h, and subsequently infected with SeV or mock for 24 h. The expression of IFN-β mRNA was measured by RT-qPCR and normalized to GAPDH ($n = 3$, biological replicates). (**H**, **I**) Promoter regions of human IFN-ω and IFN-α16. The E-box and SRE motifs are shown in the green and yellow boxes, respectively. Numbers represent the positions of the nucleotides from the start of the ORF. (**J**) The IFN-ω reporter plasmid was transfected into HeLa cells with *Renilla* luciferase plasmid (internal control). Twenty-four hours after transfection, cells were treated with pitavastatin (2 μM) at indicated h. Cell lysates were prepared, and the luciferase activities were measured and normalized to *Renilla* luciferase activities ($n = 3$, biological replicates). Data Information: In (**A–G**, **J**), data are represented as mean ± SD (****$p < 0.0001$, one-way ANOVA (**A–D**, **J**), two-way ANOVA (**E**, **G**)).

© The Author(s)

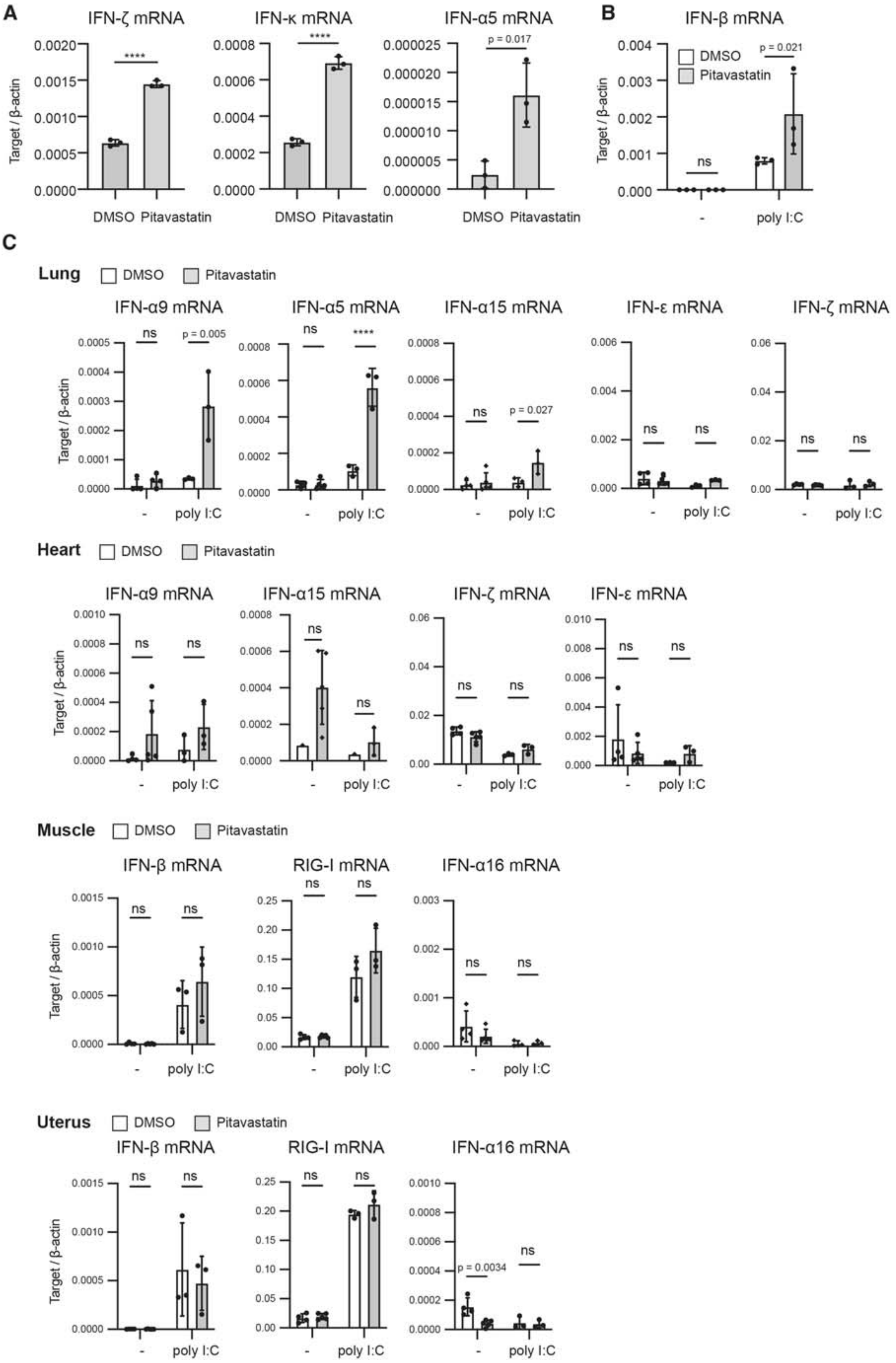

A
IFN-ζ mRNA
IFN-κ mRNA
IFN-α5 mRNA
Target / β-actin
DMSO Pitavastatin
p = 0.017
B
IFN-β mRNA
DMSO
Pitavastatin
p = 0.021
ns
poly I:C
C
Lung
IFN-α9 mRNA
IFN-α5 mRNA
IFN-α15 mRNA
IFN-ε mRNA
IFN-ζ mRNA
p = 0.005
p = 0.027
Heart
IFN-α9 mRNA
IFN-α15 mRNA
IFN-ζ mRNA
IFN-ε mRNA
Muscle
IFN-β mRNA
RIG-I mRNA
IFN-α16 mRNA
Uterus
IFN-β mRNA
RIG-I mRNA
IFN-α16 mRNA
p = 0.0034

 © The Author(s)

Figure EV5. Tissue-specific effects of pitavastatin on type I IFN responses.

(**A**) BMDMs of mice were treated with pitavastatin (10 μM) for 24 h, and the expression of noncanonical type I IFNs was measured by RT-qPCR normalized to β-actin. (**B**) BMDMs treated with pitavastatin (10 μM) for 24 h were stimulated with transfection of poly I:C (200 ng/ml) for 4 h. The expression of IFN-β mRNA was measured by RT-qPCR normalized to β-actin ($n = 3$, biological replicates). (**C**) Pitavastatin or solvent (DMSO) was intraperitoneally injected into mice every day for three days, followed by intraperitoneal injection of poly I:C (200 μg/head) or solvent (DMSO). Twenty-four hours after poly IC injection, the lung, heart, muscle, and uterus were isolated. The total RNAs were extracted from each tissue. The expression of each gene was determined by RT-qPCR and normalized to that of β-actin ($n = 3$–6 mice for each group). Data Information: In (**A**, **C**), data are represented as mean ± SD. Statistical significance was determined using t-test (**A**) and two-way ANOVA (**B**, **C**). ****$p < 0.0001$. ns: not significant.

© The Author(s)
